# Farther Account of the Case Detailed in Article Third in This Number

**Published:** 1846-09

**Authors:** 


*Farther account of the case detailed in article third in this number.*—Since the third article of the original communication was printed, the specimen referred to has been received, and was exhibited at the meeting of the Buffalo Medical Association for September. We availed ourselves of that opportunity to examine it, and can vouch for the correctness of the description given in the communication referred to, and by Dr. Hamilton in the following letter, which, by request, has been obligingly contributed. Two very interesting questions are connected with the case. It was stated by the mother of the unfortunate patient, that she had always menstruated regularly until three months before her death. Whence was the menstrual fluid derived? It does not seem possible that the lining membrane of the uterus was in any way concerned in its production, and, if not, it must have been furnished by the vaginal membrane alone. With the common ideas, as to its source naturally, it must be regarded as furnished from the latter source only by a vicarious action.

The second, and still more interesting inquiry, relates to the manner in which the seminal fluid was instrumental in producing impregnation. Our own impression is that there must have been, previous to impregnation, a minute aperture sufficient for the introduction of a sperm nuclei, although the closest scrutiny of the parts now discloses no evidence that such a communication ever existed. —Editor.

BUFFALO, Sept. 5, 1846.

DR. FLINT,—Dr. ——, (for obvious reasons names are suppressed,) a few days since brought to me the ruptured uterus and fœtus described in his letter, and in his presence, with Dr. Mixer, I have examined the whole carefully.

The uterus is torn near the attachment of the right broad ligament—walls on this side excessively thinned, so much so in some places as to leave little else than peritoneum,—opposite side of uterus about ⅓ of an inch in thickness, and of usual consistence. Length of body of uterus, 3 inches—breadth, 2½ inches; cervix uteri 2 inches long, firm and broad at base, but gradually and regularly tapering to a thin, flattened extremity, covered by a smooth mucous membrane. Upon a careful dissection of the cervix, longitudinally and transversely, no channel, or line, or cicatrix, indicating the previous existence of an os tincæ could be discovered. The structure is rather more dense than that of the uterus in its natural state, but its density is uniform—it is not scarred or corrugated, nor does it present any evidence of its being a result of disease. The mucous coat which covers it, lies free, and can be as easily removed as such membranes usually may be.

The right fallopian tube terminates at about the middle of the right side of uterus—right ovarium is larger than natural, but apparently healthy. On the left side there is no broad ligament, nor fallopian tube, nor ovarium, but the peritoneum covers it as smoothly as in front.

Dr. —— adds to what has been said in his letter, that the girl was always healthy—had menstruated 3 years, or until last 3 months, regularly.

The whole was laid before the Buffalo Medical Association for their examination, at its last meeting, (Sept. 2d,) and the records of the society certify substantially to the same as above.

Through the politeness of Dr. ——, the uterus, &c., is now the property of the Buffalo Medical College, and may at any time be seen by medical gentlemen. F. H. HAMILTON.

